# Do whale-watching experiences and tourist expectations align? A comparison of three Macaronesian destinations

**DOI:** 10.1371/journal.pone.0342997

**Published:** 2026-03-03

**Authors:** Claudia Hurtado-Pampín, Raquel de la Cruz-Modino, David Domínguez-González, Álvaro Herrera-Cáceres, Sara Vicente-Alonso, Patricia Arranz

**Affiliations:** 1 Ecología de las comunidades marinas y conservación (ECOMAR), Departamento de Biología Animal, Edafología y Geología, Universidad de La Laguna, Tenerife, España; 2 Instituto de Investigación Social y Turismo (ISTUR), Universidad de La Laguna, Tenerife, España; 3 Universidad de La Laguna, Tenerife, España; 4 Biodiversidad, Ecología Marina y Conservación (BIOECOMAC), Departamento de Biología Animal, Edafología y Geología, Universidad de La Laguna, Tenerife, España; National Cheng Kung University, TAIWAN

## Abstract

This study examines the alignment between whale-watching experiences and tourist expectations in three different destinations. Whale-watching is a global tourist activity, with locations such as the Canary Islands (Spain) and the Azores (Portugal) in Macaronesia rapidly becoming prime spots for these marine activities. Those locations attract a significant number of tourists with varying recreational interests and diverse perceptions of each destination and its natural resources, including marine wildlife megafauna species that can be seen. While often marketed as sustainable tourism, the ecological impacts of whale-watching are a matter of concern. Evolving whale-watching practices may reinforce or diminish the effectiveness of conservation and environmental education efforts. In this regard, exploring whale watchers’ expectatives, preferences, previous experiences, level of satisfaction, and environmental information may help to assess better practices and sustainable tourism initiatives. This study employed a multidisciplinary approach, incorporating a series of questionnaires that explored whale watchers’ expectations and overall satisfaction before and after sea trips in the Tenerife and El Hierro Islands (Canary Islands, Spain), as well as in São Miguel (Azores, Portugal). The findings highlight differences across three study cases: El Hierro attracted more experienced and oriented tourists, while Tenerife and São Miguel received more generalist visitors. Satisfaction was closely linked to the number of cetacean sightings. Many participants who did not mention specific species expressed open preferences such as wanting to “see everything” or “whatever is possible”, reflecting limited prior knowledge. The study highlights the importance of tailoring whale-watching strategies to tourist profiles by enhancing communication, adjusting group sizes and vessel types, and reinforcing conservation messaging to ensure both positive experiences and long-term ecological sustainability.

## 1. Introduction

The intensification of human activities in marine areas underscores the urgent need for sustainable practices that minimise ecological harm while enhancing long-term viability [1]. Marine wildlife tourism [2, 3], such as whale-watching (WW hereafter), is a growing segment that provides opportunities to observe wild animals up close in their natural habitats [4, 5]. Since its emergence in the early 1950s in Monterey Bay, California, WW has expanded worldwide and, by the 1980s, became a key component of marine tourism. Within this context, the region of Macaronesia has become one of the leading international areas for the activity, with the Canary Archipelago (Spain) established as one of the four top destinations in the world [6, 7].

WW is frequently promoted as a sustainable tourism activity or “ecotourism”. According to the United Nations World Tourism Organisation (UNWTO), ecotourism balances environmental, economic, and socio-cultural aspects to ensure long-term viability [8] and supports environmental awareness and cetacean research [9, 10]. However, its sustainability depends heavily on the effective management of the activity, involving various stakeholders, including whale watchers. While WW is a key driver of marine wildlife tourism, challenges such as increased competition among operators, pressure to reduce costs, and a focus on high visitor turnover all compromise both service quality and animal welfare [11]. Therefore, when poorly regulated, WW disturbs the animals and causes behavioural changes, adding to the multiple stressors they are exposed to [12, 13, 14, 15], which threaten their survival [16, 17].

On the other hand, tourism satisfaction is mainly based on the overall experience at the destination, where expectations and destination images play a significant role. The quality of the services, accessibility, and prior experiences may also influence satisfaction and the narrative of the destination’s reputation [11]. Thus, it is essential to ensure that whale watchers’ expectations align with the reality of the ecosystem they visit, while practising sustainable and responsible behaviour both socially and ecologically. Marketing campaigns often emphasise close encounters in pristine environments, creating unrealistic expectations that compromise both visitor satisfaction and conservation goals [11, 18]. The mismatch between expectations and reality impacts the success of WW and the welfare of the animal. For example, this can lead to harmful practices such as staying longer or closer than allowed. In summary, WW must navigate the complex balance between diverse stakeholder interests, regulations, and meeting the expectations of whale watchers without compromising the welfare of cetaceans [9, 19, 20, 21]. The effective marine management of WW should consider the expectations of whale watchers, the experiences, and blend satisfaction and the characteristics of the marine ecosystems to be preserved [11, 22].

Although guidelines are in place in some destinations to mitigate disturbance, enforcement remains a significant challenge, thus compromising the long-term sustainability of the practice [7, 23], as well as threatening whale watchers’ satisfaction [11, 24, 25]. Most research on whale watchers has focused on their satisfaction, behaviour [26], and economic impacts. However, such research often overlooks the interaction between the WW experience and environmental impact [11, 22]. To design and implement better policies and practices towards responsible and sustainable WW tourism in destinations such as the Canarian and Azorean (Portugal) Archipelagos—the two Archipelagos in the region of Macaronesia, where this study was carried out-—this research explores the relationship between whale watchers’ expectations and satisfaction, and animal welfare [5]. Accordingly, the central focus is to examine the interrelations between whale watchers’ expectations and satisfaction with their WW experiences, as well as the type of interaction they wish to establish with the animal during the trips.

Through this approach, the study contributes to the understanding of key elements—expectation, satisfaction, and intentions—underpinning responsible and sustainable WW management. Educational and interpretive strategies play a pivotal role in shaping the expectations of whale watchers and enhancing their awareness of marine conservation [9, 27]. By addressing these interconnected factors, this research also helps to fill key gaps in our understanding of WW dynamics.

## 2. Study areas

Three main WW destinations in Macaronesia were selected, considering the high popularity of WW in the Canary and Azorean archipelagos. The islands of El Hierro (EH) and Tenerife (TF) in the Canary Archipelago, and São Miguel (SM) in the Azores. Other Macaronesian archipelagos, such as Madeira, have shown some development of WW tourism, but the scale and popularity of the activity remain limited compared to the previous islands [7]. In contrast, the Savage Islands lack recorded WW activity, and it is still incipient in Cape Verde. Additionally, the maturity of the activity and the feasibility of data collection also influence the choice of the Azores and Canary Islands, which are therefore identified as the most suitable settings for this study.

**Fig 1:**
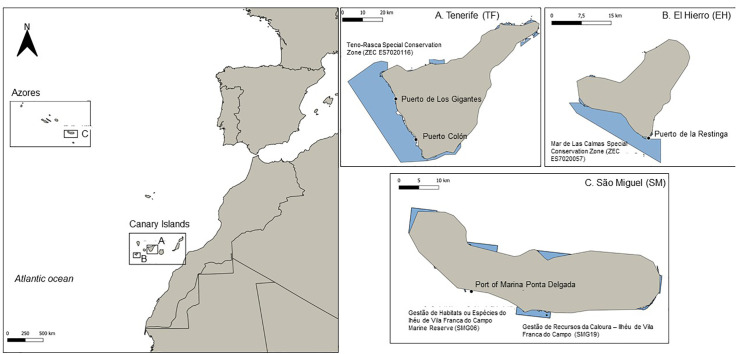
Map showing the location of the two Archipelagos, the Canary Islands and the Azores, with details of the three islands (El Hierro, Tenerife, and São Miguel), including the working ports and protected areas. Original figure created by the first author (CHP) specifically for this study. Published under a CC BY 4.0 license. © Claudia Hurtado-Pampín, 2025.

Although WW is a consolidated activity in the three islands mentioned above (Fig 1), they differ markedly in their ecological conditions (species diversity, habitat types, and oceanographic features) and socioeconomic contexts (tourism intensity, local dependence on marine activities, etc.). Additionally, the activities on these islands vary based on factors such as seasons, species, and boat size. The differences between permanent and migratory animal populations, human pressures, tourism models, seasonality, the types of WW boats used, and the regulations governing the activity all contribute to a range of WW tourism scenarios with unique characteristics, challenges, and opportunities (Table 1). The diversity observed in this context presents an opportunity for comparative analysis within a relatively uniform biogeographic region. These variations yield valuable insights that can improve marine tourism management in Macaronesia and beyond. The identified differences are crucial for understanding how WW activities evolve in different locations and under various conditions.

**Table 1:** Evaluation of key criteria across the study islands in the Macaronesian region (scale: High, Moderate, Low).

Evaluation Criteria	TF	EH	SM
**Species diversity**	High	Moderate	High
**Presence of migratory species**	High	Moderate	High
**Resident cetacean populations**	High	Moderate	Low
**Ecological integrity/ pristine habitats**	Moderate	High	High
**WW development stage/level**	High	Low	Moderate
**Scale of the WW operations**	High	Low	High
**Seasonality of WW**	Low	Low	Moderate
**Existing WW regulations**	High	High	High
**Human pressure**	High	Low	Moderate
**Presence of Mass tourism activities**	High	Low	Low
**Presence of Ecotourism activities**	Low	High	High

### 2.1. The Canary Archipelago

The Canary Archipelago is the fourth most popular WW destination worldwide and the most significant destination in Europe due to the number of available days, approximately 300 days/year [28, 29, 30, 31, 32]. The archipelago is rich in marine biodiversity and landscapes, offering the unique opportunity to see up to 32 cetacean species in their natural habitat year-round. Local coastal populations of small odontocetes include short-finned pilot whales (*Globicephala macrorhynchus*) and bottlenose dolphins (*Tursiops truncatus*), as well as larger species such as Blainville’s beaked whales (*Mesoplodon densirostris*), Cuvier’s beaked whales (*Ziphius cavirostris*) and sperm whales (*Physeter macrocephalus*). There are also seasonal visitors such as spotted dolphins (*Stenella frontalis*) and Bryde’s whales (*Balaenoptyera brydei*) [33, 34, 35]. In addition, other marine species can be observed in the area, including various species of sea turtles and elasmobranchs [36, 37, 38, 39, 40, 41].

As of the early 1990s, Tenerife (TF) emerged as a pivotal hub for WW, boasting the highest concentration within the Archipelago [7]. The southern part of TF has the highest concentration of WW operators, thanks to the consistently favourable sea and weather conditions throughout the year. This area is part of the Teno-Rasca Special Conservation Zone (ZEC ES7020116). Additionally, this island is the most veteran in terms of tourist resorts and activities designed to attract visitors. Three “S” – sun, sea and sand – tourism model [7, 42] is a significant component of the tourism industry in the area, drawing approximately 5.6 million visitors annually, 17.5% of which participate in WW activities, reinforcing the island’s position as a mass tourism destination [30]. This growth in WW activity has led to its massification, which, while economically beneficial, also raises concerns about environmental sustainability and the long-term impact on local ecosystems [27, 43, 44]. As a result, the Canary Archipelago has well-developed WW regulations. In 1995, Royal Decree 320/1995 established the first regulatory framework for WW [45], which required permits, safety, and environmental standards, as well as guides on board and guidelines for approaching the animals [46]. Updates in 2000 (Decree 178/2000) and 2007 (Royal Decree 1727/2007) further refined these measures, making them among the most comprehensive globally [47].

In comparison, the island of El Hierro (EH) has 11,423 inhabitants and fewer than 10 hotels, resulting in lower tourist volumes. In 2021, there were fewer than 250 official tourism beds, between rented holiday homes and apartments [48]. Only one official WW company currently operates in the area, with likely less impact than in other, more densely populated islands (both in resident and tourism terms). EH offers the unique opportunity to observe two beaked whale species (*M. densirostris* and *Z. cavirostris*) very close to shore [49], one of the world’s most important sites for observing these species [7, 50]. Notwithstanding, until 2019, local marine tourism companies did not carry out WW, likely due to the challenge of observing these species, which surface only briefly [51, 52]. In 2019, WW tourism initiatives were introduced through a scientific programme that prioritised sustainable practices and local engagement [7], within which the data for this study were collected. WW activities are carried out in the Mar de Las Calmas Special Conservation Zone (ZEC ES7020057), which includes Punta de La Restinga – Mar de Las Calmas Marine Reserve of Fishing Interest.

### 2.2. The Azores Archipelago

The Azores Archipelago also sees a diverse range of cetacean species, making WW a prominent component of marine tourism in the region [53, 54]. Resident populations of common (*Delphinus delphis*) and bottlenose dolphins (*Tursiops truncatus*), and sperm whales (*Physeter macrocephalus*) can be spotted year-round. Migratory species such as spotted dolphins (*Stenella frontalis*), blue whales (*Balaenoptera musculus*), Sei whales (*Balaenoptera borealis*), Bryde’s whales (*Balaenoptera brydei*), and humpback whales (*Megaptera novaeangliae)* can be observed in certain seasons. The Azores, with a 15.5% annual growth rate in WW, is one of the regions with the highest growth rates in Europe [2, 29, 35, 55]. Furthermore, other marine species, including several types of sea turtles and elasmobranchs, can be observed passing through the area [37, 39, 56]. The archipelago is rich in natural, cultural, and landscape resources, positioning itself today as a destination that promotes responsible nature tourism, in its transition from whaling past [54, 57]. The development of WW began in the 1990s, catalysed by the International Fund for Animal Welfare (IFAW), in the central group of islands in 1993 (first in Pico and then in Faial). It later expanded to São Miguel (SM), the largest island in the Archipelago, in the eastern group, in 1995. Former observers, who had previously worked for the whalers, transitioned to spotting animals for WW companies [7, 53]. Efforts towards effective WW regulation began in 1995 with the implementation of the first regional law, enforced in 1999. Subsequent modifications occurred in 2003 (DLR 10/2003/A) and the following year with “DLR 13/2004/A”. Despite the challenges posed by the increasing number of vessels and tourists, the Azores’ commitment to responsible WW practices is evident through ongoing regulatory updates and adherence to international best practices [57].

At the regional level, the Azores have protected areas, such as the Parque Marinho dos Açores. At the island level, SM has the Parque Ilha, which encompasses six Marine Protected Areas (MPAs). Many WW activities occurred in the south of the island, where different MPAs are located, such as the case of the Gestão de Habitats ou Espécies do Ilhéu de Vila Franca do Campo Marine Reserve (SMG06) and Gestão de Recursos da Caloura – Ilhéu de Vila Franca do Campo (SMG19), where the WW operators collaborating with this study carry out their WW sea trips. Unlike the Canary Islands, the tourist season in SM is limited to a few months per year due to unfavourable weather conditions and low tourism inflow in winter, which restricts the number of whale watchers and operators [53, 58].

## 3. Methodology

**Fig 2:**
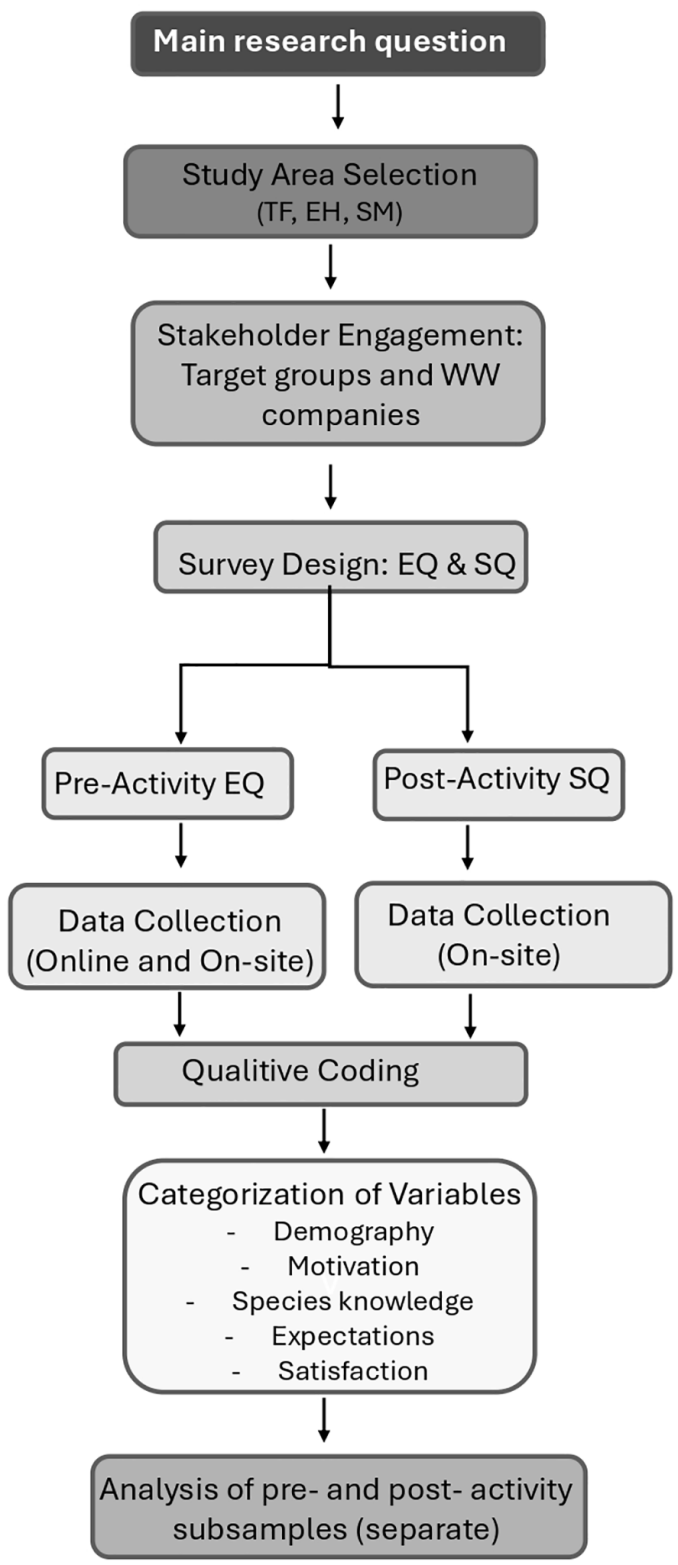
Methodological Structure of the Study across the Three Islands.

We employed a qualitative approach, utilising methods from the social sciences [11, 53, 59], including two types of questionnaires. Two different questionnaires were created to evaluate the expectations of locals and tourists participating in WW (S1 Appendix) and their satisfaction after the experience (S2 Appendix). The data collection was entirely random and voluntary. Questionnaires were conducted over various fieldwork periods between 9 January 2020 and 12 March 2023, depending on the seasonality of the activity in each destination and the availability of the WW companies. The research team consisted of marine biologists and anthropologists with a strong focus on marine social sciences, as well as natives and/or permanent residents from different Canary Islands, and had experience in WW tourism in the Azores. Additionally, interaction with the WW companies was key to planning and developing the study, for example, selecting languages for the questionnaires and determining whether they could be delivered online (Fig 2).

### 3.1. Pretest and ethical standards

Both questionnaires were initially designed and tested in EH within the project *Technification and sustainability of whale-watching and maritime transport in the Canary Islands: optimising the detection and observation of cetaceans with implications for their conservation*, reviewed and approved by the area of innovation of the Island Council. Later, the Government of the Canary Islands also reviewed the study through the Agency for Research, Innovation and the Information Society, part of the Regional Ministry of Universities, Science, Innovation and Culture. Subsequently, a second proposal, titled *“Identification of Synergies for the Development of Blue Tourism in Natura 2000 Network Sites.”* was approved, enabling the expansion of research activities to other Macaronesian study sites until 2023. Additional permits for fieldwork and data collection were not required.

Expectation (EG) and Satisfaction Questionnaires (SQ) were designed to be completed either autonomously by the whale watchers or with the assistance of a trained interviewer. They included both closed-ended (quantitative) and open-ended (qualitative) responses, and were anonymous (Fig 2). All participants were adults. At the beginning of the participation, respondents read a disclaimer of liability included in the questionnaire’s models (see S1 and S2 Appendix). Informed consent was obtained verbally; no personal data was collected, and the results remain confidential and have been used for research purposes only. This procedure adheres to the University of La Laguna’s (ULL) data protection policy and complies with Spanish Laws. The final dataset was approved and published in the ULL Research Database, an open data repository that promotes the use of FAIR (Findable, Accessible, Interoperable, Reusable) data, in accordance with the European Union’s recommendation for the publication of scientific data and was used to conduct the retrospective analysis.

### 3.2. Data sampling

We adapted Oram’s approach [19] to allow for independent samples for the assessment of pre (expectations) and post (satisfaction) WW activity perceptions and behaviours, rather than surveying the same individuals before and after the activity. In tourism literature, it is common to use independent pre- and post-samples (e.g., arriving vs. departing) when tracking the same individual is not feasible [60, 61]. Thus, the study employed a pre-post design with independent individuals but sharing the same trip, collecting data at two points in time: at the beginning of the WW trip (measurement of expectations) and at the end of the WW trip (measurement of satisfaction). The adjustment was necessary due to logistical constraints in the field.

Specifically, data collection was carried out in collaboration with WW and marine tour companies, as mentioned above, subject to their availability and operational schedules. In this context, linking individual responses was not feasible due to scheduling and operational limitations of the participating companies. On the other hand, the pre-activity questionnaires were administered mainly online and anonymously to maximise participation and comply with ethical standards, whereas the post-activity questionnaires were completed on-site during the WW trips. Therefore, the pre-activity online and anonymous questionnaires made it impossible to match responses with those of the post-activity respondents.

To mitigate this limitation, we ensured that both pre- and post-activity samples were sufficiently large and participants were randomly selected, allowing for reliable and valid comparisons between the two groups. Additionally, only separate comparisons were performed between the pre- and post-activity subsamples, without conducting descriptive or statistical tests between the two samples within the same destination. This approach was adopted to avoid potential biases, as pre- and post-activity samples differ in terms of national backgrounds, prior expectations, or travel types, which could lead to variations in averages that do not strictly reflect the effect of the WW experience.

### 3.3. Questionnaires and variables

#### 3.3.1. Questionnaires.

The Expectation Questionnaire (EQ) was designed to be completed online before engaging in the activity, upon confirmation of booking, or before boarding the WW boat at each location. The companies distributed the questionnaire via email, primarily using the Google Forms platform (S1 Appendix); no email addresses or identifying information were collected. Due to challenges in obtaining responses, additional on-site trained interviewers facilitated questionnaire completion for participants waiting to board at ports or centres.

The WW participants in TF boarded in Puerto Colón and Puerto de Los Gigantes, both located in the south of the island. Here, field interviews also benefited from the collaboration of the harbour involved, as well as the Tenerife Tourism Board (Turismo de Tenerife), a state entity dependent on the Island Council, which promoted collaboration with the WW companies. Although in EH, EQ was distributed online by the WW company or at the reception before the activity using a QR code, complimentary field interviews were also conducted in Puerto de La Restinga, the island’s leading marine tourism destination. Similarly, in SM, whale watchers boarded at the port of Marina de Ponta Delgada.

In contrast, Satisfaction Questionnaires (SQ) were completed immediately after the WW trip, typically aboard the vessel during the return journey, with support from the interviewers (S2 Appendix). This questionnaire was designed to be shorter, focusing on direct inquiries regarding the experience due to time constraints.

The EQ comprised 11 questions in four sections (Table 2). The first section gathered demographic information, travel characteristics, and primary motivations to obtain a comprehensive profile of the participants. In the following section, respondents were asked about their preferences and expectations regarding species sightings, the perceived importance of these encounters, and their expectations for interactions with animals. Also, they were asked about any previous WW experiences. Finally, participants were asked if they had received sufficient information about the activity’s characteristics prior to the WW trip.

**Table 2:** Sections and Indicators of the EQ with Corresponding Question Numbers.

Expectation Questionnaire		
SECTION	INDICATORS	QUESTION NUMBER
1. Participant Demographics and Motivations	Demographics	1, 2, 3, 4
	Travel motivations	5
2. Previous WW Experiences	WW Experiences	6
3. Species Preferences and Expectations	Species preferences	7
	Species expectations	8
	WW activity expectations	9, 10
4. Information before the activity	Level of prior information	11

The SQ had seven questions organised in four sections, including a brief description of the whale watchers’ participants’ origin and previous WW experience. Participants were asked about the species they initially expected to see and those finally observed, as well as their overall satisfaction with the experience and prior knowledge about the activity (Table 3).

**Table 3:** Sections and Indicators of the SQ with Corresponding Question Numbers.

Satisfaction Questionnaire		
SECTION	INDICATORS	QUESTION NUMBER
1. Participant Demographics	Demographics	1, 2, 3
2. Previous WW Experiences	WW Experiences	4
3. Species Expectations and Observations	Species expectations	5
	Species observations	6
4. Information about their Experience	Satisfaction	7

#### 3.3.2. Variables.

The structure and content of both questionnaires were developed to address the study’s primary objective of examining the relationship between whale watchers’ expectations, satisfaction, and perceptions of animal welfare. Each question was designed to capture variables identified in previous literature as relevant to visitor experience and sustainability in WW [5, 11, 19]. The sections on species preferences and expected interactions allow for an assessment of the alignment between visitors’ expectations and ecological realities. At the same time, questions on satisfaction and information received provide insights into the role of interpretation and management in shaping post-experience evaluations. Together, these variables enable an integrated understanding of how pre-trip expectations and on-site experiences influence satisfaction and, ultimately, the sustainability of WW practices.

Demographic and motivational variables provided context for visitor profiles. Expectation-related variables (e.g., preferred species, desired proximity, and prior information received) were used to evaluate pre-trip perceptions and knowledge. Satisfaction variables (e.g., observed species, overall experience, and adequacy of information) were used to assess post-trip evaluations. These variables collectively enabled the exploration of how visitor profiles and expectations shape satisfaction and perceptions of sustainable WW practices (Table 4).

**Table 4:** Summary of variables used in the analysis.

VARIABLE	DESCRIPTION	SOURCE QUESTION	TYPE	PURPOSE IN ANALYSIS
Country of origin	Region of participant’s residence	EQ Q1	Categorical	Identify visitor profiles
Motivation	Main reason for the trip	EQ Q5	Categorical	Understand travel purpose
Species expected	Species participants wanted to see	EQ Q8	Categorical	Assess expectation specificity
Desired interaction	Preference for proximity vs. distance	EQ Q9	Categorical	Evaluate wildlife interaction attitudes
Information before the trip	Self-assessed adequacy of information	EQ Q11	Ordinal	Measure awareness and preparation
Satisfaction	Overall satisfaction after the trip	SQ Q7	Ordinal	Measure post-experience evaluation

### 3.4. Companies and vessel profiles

Seven companies were involved in this study: five in TF, one in EH, and one in SM. The companies offered trips of four hours or less, and some of them included complementary elements, such as food and beverages on board or sea bathing. All the companies had on-board guides, not necessarily with specialist certification, to provide specific information about the marine wildlife observed.

There were two distinct types of WW vessels in TF. One type was a fibreglass inflatable vessel or sailboat, measuring approximately 10–12 meters in length, with a maximum capacity of 12 people; the other was a 17.5-meter catamaran, accommodating over 70 participants. The analyses were performed separately for each type: TF1 for the small-vessel experience and TF2 for the large-vessel experience.

In EH, the study was conducted on an inflatable vessel measuring 9.5 m in length, with a maximum capacity of 12 participants.

In SM, the study was conducted on a 17.5 m catamaran with a capacity of 70 participants, similar to the one used in TF2.

### 3.5. Data processing

For the processing of qualitative responses from both questionnaires, an adapted grounded theory approach was employed. This method allowed the identification of key interpretative categories emerging from the participants’ open-ended responses. The process followed a structured three-stage coding strategy: open coding was first applied to identify recurrent words and expressions; axial coding was then used to organise these codes into broader conceptual categories; and finally, selective coding helped identify core thematic dimensions that were cross-cutting among participants. Coding was conducted manually and independently by at least two co-authors for cross-validation purposes [62, 63] (Fig 2).

The codification of the species knowledge for both questionnaires, including species preferences, expected sightings, and reported sightings, was grouped into three categories: ‘No knowledge,’ ‘Average-knowledge,’ and ‘Broad-knowledge.’ These groups were based on whether the species mentioned by respondents are commonly observed on the destination, as proposed by Sousa et al. [35, 64]. When the respondent did not specify a particular cetacean species but used general terms like “whale” or “dolphin” in their answers to corresponding questions, categories such as “Generic Dolphin” and “Generic Whale” were created for analysis. Conversely, when respondents named a particular cetacean species, that species was used as a category, and all these answers were classified as “Specific dolphin species” or “Specific whale species”. Furthermore, for analytical purposes, participants’ countries of origin were grouped into five regional categories: Atlantic Europe, Central Europe, Mediterranean Europe, European Iberia and Outside Europe [65]. This classification was established to account for potential regional differences in travel patterns and preferences (Fig 2).

Following data compilation and coding, statistical analyses were conducted using IBM SPSS v.29 [66] to perform cross-tabulation analyses and investigate the relationships between variables, as well as to establish the frequencies of specific categories for each variable. To assess the significance of the association, we employed Pearson’s chi-squared test [67], suitable for detecting associations in large sample sizes with sufficiently high expected frequencies in each contingency table cell. We also used Fisher’s exact test due to the presence of variables with expected frequencies lower than 5, which may violate the assumptions of Pearson’s test [68]. Fisher’s test is more suitable when dealing with low expected frequencies or small sample sizes, as it provides more accurate results under these conditions.

Additionally, cross-tabulation analyses were conducted for variables with multiple responses. The study examined the relationships between one or more categorical variables and those with various response categories, allowing for the exploration of patterns and associations in complex datasets where respondents may select various options. Likewise, we analysed variance (ANOVA) using Tukey’s post-hoc test [69] in RStudio v 4.2.3. [70] to examine the relationship between the variables ‘destination island’ and ‘satisfaction.’ This method enabled pairwise comparisons between the means, identifying significant differences in satisfaction level across islands while accounting for multiple comparisons and controlling the familywise error rate. ANOVA was deemed appropriate for assessing the dependent variable of satisfaction in relation to the independent variable ‘destination island’, in line with the study’s objectives.

The approach employed, combining qualitative coding with cross-tabulations, chi-square/Fisher’s tests, and ANOVA with post-hoc comparisons, aligns with the exploratory and comparative nature of the study. These methods enabled the testing of initial hypotheses without compromising the interpretation of results. Therefore, additional segmentation or regression analyses were deemed unnecessary for the study’s objectives.

## 4. Results

In total, 476 EQ and 347 SQ surveys were collected across the three islands, revealing significant differences in participant characteristics, expectations, and satisfaction levels (Table 5).

**Table 5:** Total number (n) of EQ and SQ conducted across the three island destinations.

Archipelagos	Island	Total of EQ (n)	Total of SQ (n)
Canary Islands	El Hierro	120	81
	Tenerife	142	173
Azores	São Miguel	214	93

### 4.1. Participant profile by destination island

**Fig 3:**
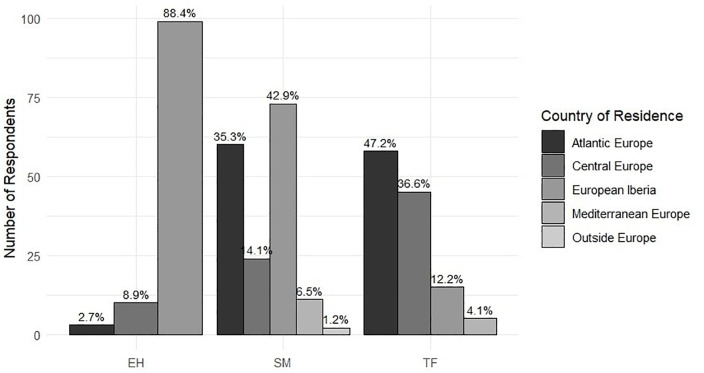
Number of respondents per island by country of residence, with percentage of each country within the island shown above bars.

According to the EQ results, a significant association was found between participants’ country of origin and destination island, as determined by a chi-squared test (df = 12, N = 436, p-value < 0.001). Most participants in EH (88.4%) and SM (42.9%) were from European Iberia, whereas in TF, the majority came from Atlantic and Central Europe (47.2% and 36.6%, respectively) (Fig 3).

**Fig 4:**
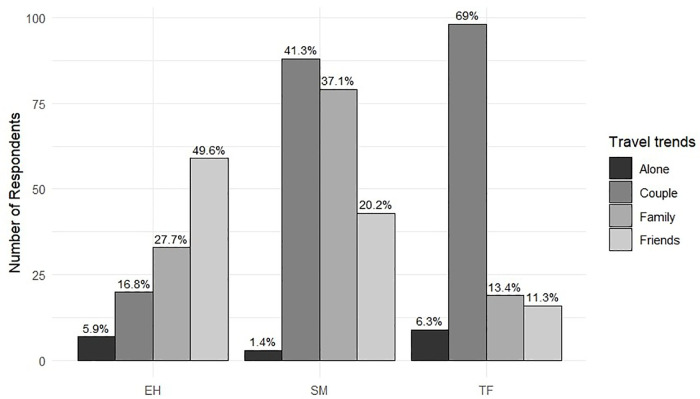
Number of respondents per island by travel group, with percentage of each group within island shown above bars.

Travel group composition also differed significantly across destinations (chi-squared test, df = 6, N = 474, p-value < 0.001). In TF, most participants travelled as couples (69%), while in SM, this group was also predominant (41.31%). In EH, the most common group was friends (49.6%). Unaccompanied travellers were a minority in all cases (6.3% in TF, 1.4% in SM, and 5.9% in EH) (Fig 4).

**Fig 5:**
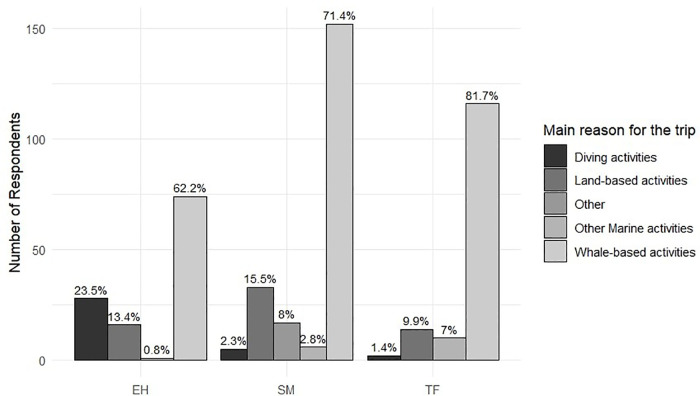
Number of respondents per island by main reason for the trip, with percentage of each reason within island shown above bars.

Regarding the primary motivation for travel, significant differences were again observed among destination islands (chi-squared test, df = 8, N = 474, p-value < 0.001). Whale-based activities were the primary reason in TF (81.7%) and SM (71.4%), while in EH. However, still the majority (62.2%), scuba diving (23.5%) and land-based activities (13.4%) were more frequently reported as alternative motivations. (Fig 5).

### 4.2. Expectations before the activity

**Fig 6:**
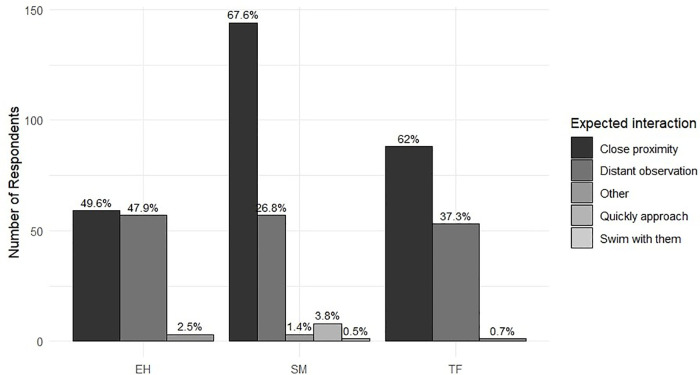
Number of respondents per locality by expected interaction, with percentage of each interaction type within island shown above bars.

All the questionnaires placed a unanimous emphasis on the significance of animal observation. Participants’ expectations about interaction with animals also varied significantly across destination islands (Fisher’s exact test, N = 474, p-value < 0.001). In TF and SM, the majority expressed a preference for a close contact (62% and 67.6%, respectively). In comparison, in EH, the responses were more evenly split between those who preferred proximity (49.6%) and those who preferred observation from a distance (47.9%) (Fig 6) (Table 6).

**Table 6:** Comparative summary of key variables across islands.

VARIABLE	EH	TF	SM	p-value/Test
% of respondents with prior WW experience	35.6%	42.3%	39.8%	χ², p < 0.05
% expecting close interaction	49.6%	62.0%	67.6%	Fisher, p < 0.001
Main species expected (whales/dolphins)	30.3/73.1	16.9/69.7	38.0/90.1	χ², p < 0.001
Satisfaction level (%)	78.6%	97.8%	95.9%	ANOVA, p < 0.05
% reporting insufficient info	45.9%	18.9%	29.1%	χ², p < 0.001

Species preferences revealed statistically significant differences between destination islands (chi-squared test, df = 6, N = 474, p-value < 0.05). In SM and TF, participants predominantly expressed ‘Generic Whales’ preferences (60.8% and 48.2%, respectively), while in EH, this preference was lower (30.4%) and a more diversified interest was noted, including a greater mention of “all species” (7%) and specific interest in beaked whales.

Species preferences for observation also vary significantly depending upon the respondent’s mode of travel (as in accompaniment) (Fisher’s exact test, N = 474, p-value < 0.05). Respondents travelling with groups of friends or family more frequently responded ‘Generic dolphin’ (43.6% and 45.3%, respectively), whereas those travelling alone (22.2%) or as couples (35.6%) mentioned this category less often. Conversely, individuals travelling alone or with their partners showed a greater inclination towards specific dolphin species (44.4%; 31.2%) as compared to those travelling with their family (21.4%) or groups of friends (29.9%). Furthermore, distinctions were noted in respondents expressing interest in specific whale species, with 22.2% of solo travellers selecting this option, compared to lower percentages among those travelling as couples (4.4%), with family (7.9%), and with friends (11.1%).

**Fig 7:**
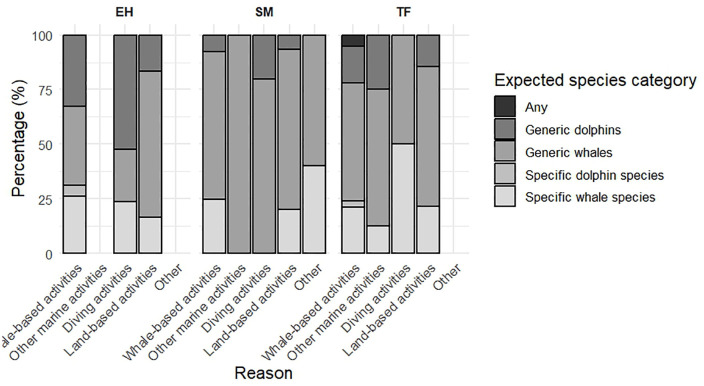
Expected species for observation by destination island and main reason of the trip.

Respondents whose primary purpose was ‘Whale-based activities’, ‘Land-based activities’, and ‘Other’ indicated ‘Generic whales’ by over 50.9%, whereas respondents with other main reasons for their trip did not (‘Other marine activities’ 20% and ‘Diving activities’ 22.6%). Conversely, those engaged in ‘Diving activities’ tended to specify species of whales (19.4%) and dolphins (41.9%) more than other groups. A stratified analysis reveals that these differences are primarily evident on EH, rather than on other islands. Additionally, it is worth noting that respondents whose main motive for the trip were ‘Other marine activities’ more frequently selected ‘Any’ (26.7%) compared to those opting for ‘Whale activities’ (9.7%), ‘Diving activities’ (12.9%), ‘Land-based activities’ (9.5%), and ‘Others’ (5.9%). Moreover, the differences in species preferences for observation were noteworthy but not significant depending on the main reason for the trip (Fisher’s test, N = 474, p-value) (Fig 7).

There were also significant differences in the species participants expected to observe depending on the island visited (chi-squared test, df = 6, N = 474, p-value < 0.001). Respondents shared anticipated dolphin sightings across all the islands. However, the expectation was more marked in SM (90.1%), followed by EH (73.1%) and TF (69.7%). Additionally, the anticipation of whale sightings is more frequent in SM (38%) and EH (30.3%) as compared to TF (16.9%) (Table 6). Striking differences were to be observed regarding anticipated beaked whale sightings, with a substantially higher percentage on EH (32.8%). The response ‘Any’ was also more frequent in TF (33.8%) and EH (23.5%), as compared to SM (9.4%).

Perceptions of the information received prior to the activity differed significantly across destinations (chi-squared test, df = 8, N = 336, p-value < 0.001). Participants from EH reported the highest proportion of unmet informational need (45.9%), followed by SM (29.1%) and TF (18.9%) (Table 6).

Additionally, in the qualitative open-ended responses, when participants were unable to specify particular species, recurring verbatim expressions emerged that reflected broad, inclusive expectations, such as “I want to see everything,” “the more the better,” “Any,” and “anything possible.” Regarding data saturation in the qualitative component, the repetition of key phrases and categories became evident early in the coding process, indicating that saturation was reached for the exploratory aims of this study. This supports the robustness and methodological transparency of the qualitative data interpretation within the mixed-methods framework employed.

### 4.3. Post-activity satisfaction

**Fig 8:**
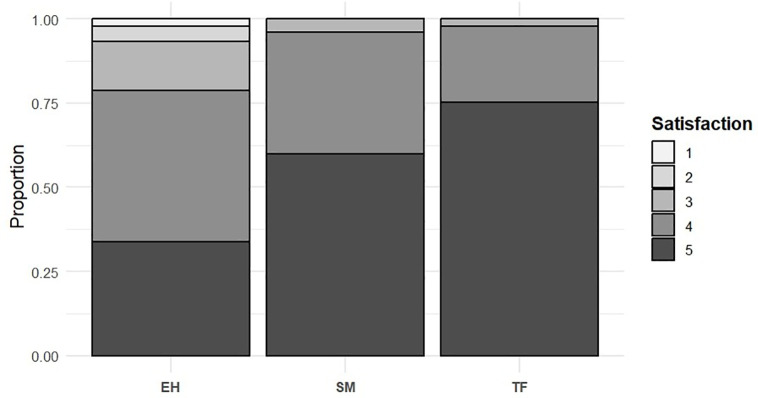
Variation in Satisfaction by Participant Destination.

Satisfaction after the activity was generally high across all islands, though significant differences were found in satisfaction levels (Tukey Test, df = 12, N = 355, p-value < 0.05). TF and SM showed the highest satisfaction rates (97.78% and 95.9%, respectively), while EH had lower satisfaction levels (78.65%) and the highest proportion of dissatisfied participants (6.7%) (Fig 8) (Table 6).

Participants’ satisfaction significantly varied depending on whether they had encounters with animals, with satisfaction levels diminishing rapidly when such encounters did not occur (50%). Observing whales, dolphins, or turtles corresponded with higher satisfaction, while the absence of sightings —more common in EH— corresponded with reduced satisfaction. A cross-tabulation analysis involving the variables ‘Observed Species,’ ‘Destination Island,’ and ‘Satisfaction’ revealed that differences in satisfaction levels were primarily attributed to the island of EH as compared to the other two islands. This discrepancy arose because EH was the destination where participants most frequently reported not observing any animals during the activity.

**Fig 9:**
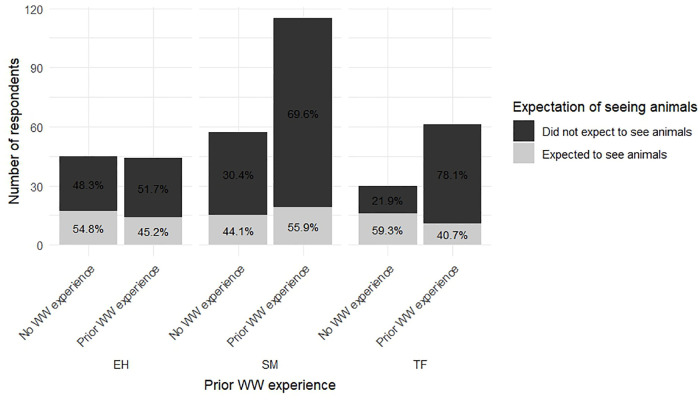
Expectations of seeing whales as a function of prior WW experience.

No significant differences were found in satisfaction levels between first-time participants and those with prior experience. However, a significant association was observed between prior WW experience and expectations of seeing whales (chi-squared test, df = 8, N = 355, p-value < 0.05) (Fig 9) (Table 6).

**Fig 10:**
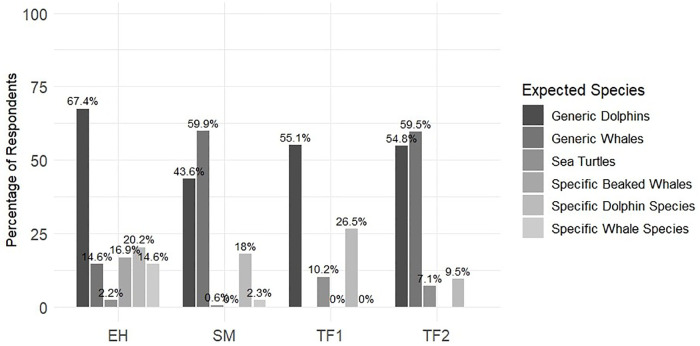
Expected species from post-activity WW trip by locality.

Significant differences were observed among the species respondents expected to observe. ‘Generic Whales’ were less frequently expected in EH (14.9%) than in other locations (TF2 59.5% and SM 59.9%). Conversely, ‘Generic Dolphin’ responses were less common in SM (43.6%) as compared to the other locations (TF1 55.1%, TF2 54.8%, EH 67.4%). Notably, ‘Sea turtles’ were more frequently anticipated in TF (TF1 10.2% and TF2 7.1%) than in other locations (EH 2.2% and SM 0.6%), and specific species of beaked whales were exclusively anticipated in EH. Furthermore, it is worth mentioning that more particular species of dolphins were anticipated in TF1 (26.5%) and EH (20.2%) by small vessels compared to large vessels (TF2, 9.5%, and SM, 18.0%). Regarding specific whale species, 14.6% of respondents mentioned them in EH and 2.3% in SM, while no responses were recorded in TF. The differences in participants’ knowledge and expectations of the species they wished to see were statistically significant among the islands (chi-squared test, df = 4, N = 355, p-value < 0.05) (Fig 10).

Finally, more specific expectations (e.g., naming specific species) were more frequent among solo travellers and couples, especially in EH and among participants on small vessels in TF, indicating a more informed or specialised interest profile.

## 5. Discussion

This study examined the relationship between expectations, preferences, and satisfaction levels across three different WW destinations in the Macaronesian Region, considering three ecologically and socially distinct islands (EH, TF and SM). The findings revealed notable variations among islands, as the activity is shaped by multiple ecological, social, and experiential dimensions that influence the WW activity and, ultimately, the sustainability of the practice.

A key finding relates to the differing national origins and group composition of the participants across the islands. These differences influence travel motivations and expectations for engaging in activities. TF and SM attracted a higher proportion of couples (69% and 41.31%, respectively) and visitors primarily motivated by WW (81.7% and 71.41%, respectively). In contrast, EH exhibited a broader diversity of motivations (62.2% for WW, 23.5% for scuba diving, and 13.4% for land-based activities) as well as a higher number of groups of friends (49.58%). Understanding these whale-watcher profiles is crucial for tailoring communication and product design, aligning with expectations and thereby enhancing experiences and promoting sustainable tourism, as outlined in the nature-based tourism literature [2, 10, 19, 26, 71, 72, 73]. The concentration of couples in TF and SM may indicate both marketing strategies emphasising romantic or short-stay experiences and the logistical ease of joining WW trips for two. In contrast, the predominance of friend groups in EH could indicate that this destination appeals to more adventurous or socially oriented tourists, suggesting that motivation is strongly tied to both social and environmental contexts.

Vessel type also influenced participants’ characteristics in TF. Large vessels tended to carry fewer environmentally engaged whale watchers, whereas smaller vessels offered more personalised experiences to participants who had a stronger conservation orientation. These results are consistent with Orams’ [19] finding, which links personal and educational experiences with pro-environmental behaviour. On islands with lower visitor volumes (EH and SM), participants showed more nature-focused motivations; particularly in SM, where WW was the primary driver (71.4%), and in EH, where WW was associated with scuba diving and land-based nature activities (62.2% for WW, 23.5% for scuba diving, and 13.4% for land-based activities). These findings suggest that vessel scale and management model shape not only the type of experience but also the visitor’s environmental engagement. Smaller vessels may foster stronger personal interactions and environmental education, creating opportunities for meaningful interpretation. Conversely, larger vessels, while economically profitable, risk diluting educational content in favour of a more commercial approach.

Whale watchers’ profiles also shaped expectations. All participants valued marine encounters, yet the degree of species specificity and desired proximity varied. TF and SM participants generally expected close encounters with the animals (62% in TF, 67.6% in SM, and 49.6% in EH). In contrast, EH participants tended to express more specific interests, such as observing beaked whales (32.8%), reflecting higher environmental awareness and prior experience. The diversity of expectations among destinations may be shaped by how WW is communicated. Marketing that emphasises close contact can set unrealistic expectations about physical proximity, leading to visitor frustration when regulations limit the distance they can approach. These patterns underscore the importance of aligning promotional messages with ecological realities and reinforcing interpretive briefings that explain the reasons behind distance and behavioural guidelines.

Preferences for wildlife marine species further reinforce destination-specific profiles. In TF and SM, participants expressed broader interest in generic whale sightings (48.2% and 60.8%, respectively), while those in EH showed inclination towards particular species and a greater appreciation for observing all marine species. Additionally, participants who identified scuba diving as their primary motivation for travel were more likely to specify cetacean species (61.3%), indicating that prior experience and environmental familiarity play a role in shaping wildlife expectations [73].

Travel group composition also played a role: unaccompanied whale watchers and couples expressed greater interest in specific species (e.g., 44.4% and 31.2% specified a dolphin species, and 22.2% and 4.4% a whale species, respectively), while larger groups of friends or families more often selected the “generic” category (43.6% and 45.3%, respectively), with lower rates of species specific mentions. This finding supports the need for visitor experience management research, which suggests that smaller group travel is associated with a more substantial interest in learning and greater receptivity to interpretive content [74]. These imply that group composition influences learning potential and engagement, a critical consideration for designing educational interventions and interpretation strategies onboard. Personalised experiences tend to correlate with more knowledgeable and engaged whale watcher profiles [7, 9, 27, 75].

Participants in this study with prior WW experience tended to express greater anticipation of seeing whales compared to first-time whale watchers, likely influenced by previous positive experiences. However, their satisfaction levels did not differ significantly from those of first-time whale watchers, suggesting that prior experience influenced expectations regarding interaction but did not influence satisfaction. These findings indicate that prior experience may shape expectations, but not necessarily satisfaction.

Our results align with previous research [75], suggesting that proximity to animals is a key factor in satisfaction. However, such expectation can be problematic, as close approaches may disrupt natural behaviour, cause stress, and interfere with navigation [23, 76]. In this context, the mismatch between marketing promises, existing regulations and ecological realities [11, 18, 75] may result in visitor dissatisfaction. Awareness-raising and education, both before and during tours, are crucial in mitigating unrealistic expectations and reducing environmental pressure. Protected marine areas, when well-managed, can support this dual goal of conservation and meaningful experiences [1]. In this context, our findings underscore the potential of MPAs, particularly those under the Natura 2000 framework, as platforms for sustainable tourism [77]. Expectation management, supported by environmental education and compliance with regulations, is central to sustainable WW tourism.

While satisfaction was generally high among the participants in this study, it varied significantly across destinations. TF and SM participants reported the highest satisfaction, associated with higher animal observation rates (91% and 99% of trip sightings, respectively), consistent with the existing literature, which highlights sightings as key drivers of satisfaction [2]. In contrast, lower sightings in EH (64% of trips) correlated with lower satisfaction, underlining the importance of aligning whale watchers’ expectations with the realities of the activity.

Furthermore, the perceived lack of information — particularly in EH — also influenced satisfaction. Educational and interpretive content, before and during the experience, can buffer disappointment from low sighting rates. Satisfaction is also shaped by emotional connection and perceived authenticity [78]. Ziegler et al. [59] and García & Pacheco [9] emphasise that conservation information is often insufficient. However, when whale watchers understand the ecological context, they may be more likely to support responsible practices [11, 19, 79, 80, 81, 82, 83]. Therefore, well-integrated messaging can foster both satisfaction and environmental stewardship [11, 72, 84, 85].

Aligning expectations around biodiversity knowledge and ecosystem interpretation, rather than guaranteed sightings, can enrich the WW experiences. Emphasising the value of marine biodiversity and conservation, along with local cultural elements, can strengthen emotional engagement. Tools such as pre-trip briefing, interpretive materials, and onboard guides contribute to this alignment [7, 9, 27, 43, 75]. Well-informed whale watchers often report higher satisfaction even when sightings are fewer [2, 9, 43].

Operators must strike a balance between conservation and visitor satisfaction [9, 19, 20, 21]. Strengthening eco-friendly practices and enforcing efficient regulations are crucial steps towards achieving this balance [2, 11, 18, 59, 86]. Successful models, for example, in Iceland, where WW companies collaborate with research centres, demonstrate how partnerships between tourism and conservation sectors can lead to more sustainable practices that benefit wildlife and the local economy [87]. In EH, where the WW emerged, supported by scientists and benefiting from collaboration between them and tourism operators, we found that 20.7% of EH participants expected to see specific dolphin species, compared to only 10.8% in TF and 19.2% in SM.

Furthermore, smaller vessels in EH also had a higher percentage of participants (14.9%) expecting specific whale species, whereas in SM, this was much lower (2.4%), with no response in TF. In contrast, large vessels tended to attract participants with more general expectations and lower engagement with sustainable WW practice. These results suggest the need for differentiated products that align with both whale watchers’ profiles and conservation goals [88] in different WW destinations.

Comparing our results with previous studies, key satisfaction drivers in WW include the number of sightings, vessel type, and cost [2, 6, 86, 88, 89]. While Orams [19] suggested that satisfaction may occur even without sightings, we still consider that an encounter is crucial for satisfaction. Our observations align with those of Duffus & Dearden [90], Valentine et al. [91], and Kessler et al. [80], which emphasise the strong link between wildlife encounters and whale-watcher satisfaction. Similarly, Bentz et al. [2] suggest that the consistently high satisfaction levels reported in the Azores are primarily attributed to the region’s near-guaranteed sightings. Further studies considering other aspects, such as the guides’ skills or complementary activities and information on board that enrich the experience, will be desirable in this field.

Whale watchers’ behaviour remains complex, presenting challenges for better management [92]. In our study, TF accommodates higher volumes of visitors with broader motivations, while SM and EH tend to attract more specialised segments, as reflected in their dominant travel motivations. These differences indicate that strategies tailored to each destination are essential; what is effective in TF may not be successful in EH or SM due to variations in visitor demographics, expectations, and environmental conditions.

The results of this study also reveal how the different tourist profiles observed across the islands influence perceptions of marine wildlife and the sustainability of WW practices. More experienced and environmentally aware participants, particularly in EH and SM, tended to value observation and species diversity over close interaction, reflecting higher conservation-oriented attitudes. In contrast, the predominance of generalist and short-stay visitors in TF, primarily motivated by proximity encounters, may contribute to greater environmental pressure and lower awareness of conservation principles. These differences underscore the necessity for adaptive management strategies that account for the distinct characteristics of tourist segments in each destination and foster conservation-oriented behaviour through education, awareness campaigns, and incentive mechanisms.

At the same time, the findings have relevant implications for policy and conservation. The concentration of mass tourism models and larger vessels in TF raises concerns regarding noise pollution, behavioural disturbance, and potential habitat degradation, as observed in similar contexts worldwide [16, 17, 23, 44]. Such patterns could have long-term ecological impacts on local cetacean populations if not properly managed. Therefore, it is essential to strengthen the enforcement of approach-distance rules, limit vessel numbers, and prioritise quieter propulsion technologies to mitigate acoustic impacts [76, 87]. As highlighted by Orams [19], Mayer et al. [93], and Scuderi et al. [94], sustainable governance frameworks should integrate conservation education, operator certification systems, and economic incentives for environmentally responsible practices. Public–private collaborations and community-based management schemes, as successfully implemented in Mexico and Iceland [93, 95], can also improve data exchange, compliance, and stakeholder engagement. These initiatives are fundamental to safeguarding marine ecosystems, enhancing visitor satisfaction, and positioning the Macaronesian region as a model for responsible marine ecotourism.

Despite the limitations, the current study design offers valid and robust insights into the patterns of expectations, experiences, and satisfaction across the three islands. Future research that incorporates linked pre- and post-activity data could enhance our understanding of how prior expectations impact satisfaction outcomes. To establish a correlation between expectations and satisfaction, methods such as assigning anonymous participant codes to connect pre- and post-activity responses, conducting longitudinal follow-ups with participants, or selectively surveying individuals both before and after the trip could be utilised. These strategies facilitate individual-level analysis while preserving anonymity and encouraging widespread participation.

## 6. Conclusions

Building on the results presented and discussed above, this study shows that WW experiences differ markedly across three Macaronesian destinations with distinct socio-ecological and tourism contexts. By comparing EH, TF, and SM, the findings demonstrate that WW experiences are not homogeneous and are strongly shaped by destination characteristics, tourism models, and visitor profiles. Rather than being interpreted merely as a recreational activity, WW needs to be analysed in light of the complexity of the socio-ecological system in which it is embedded, where tourist expectations, activity management, and conservation outcomes are deeply interconnected.

From a theoretical perspective, this research contributes to the literature on nature-based and wildlife tourism by empirically demonstrating that expectations and satisfaction are mediated by contextual factors, including vessel type, operational scale, and destination maturity. The distinction observed between generalist expectations (e.g., “seeing any whale”) and more specific species-oriented expectations provides a useful conceptual framework for understanding different levels of environmental knowledge and engagement among whale watchers. By combining qualitative and quantitative approaches, the study captures both measurable satisfaction outcomes and the meanings participants attach to their experiences. Although the use of independent pre- and post-activity samples limits causal inference at the individual level, the comparative multi-site design offers robust evidence of structural patterns that shape WW experiences across different contexts.

From a practical and managerial standpoint, the findings emphasise the importance of tailoring WW management and communication strategies to the specific profiles of visitors and the ecological realities of each destination. Satisfaction remains closely linked to animal sightings; however, the lower satisfaction levels observed in destinations with fewer encounters underscore the need for effective expectation management. Providing accurate pre-trip information, improving onboard interpretation, and clearly communicating the inherent uncertainty of wildlife encounters can help reduce frustration and prevent practices that may compromise cetacean welfare. Vessel type also emerged as a key management variable: smaller vessels attracted more specialised and environmentally engaged visitors, while larger vessels were associated with more generalist expectations. These results suggest that differentiated products, interpretive strategies, and capacity management could improve both visitor experiences and conservation outcomes, as well as cetacean welfare.

At broader social and conservation levels, this study underscores the role of WW as a platform for environmental awareness and perceptions of welfare. More experienced and specialised participants—particularly in EH and SM—tended to value observation, species diversity, and respectful distance over close interaction, reflecting stronger conservation-oriented attitudes. In contrast, mass-tourism contexts, such as TF, concentrated higher numbers of visitors with proximity-oriented expectations, which may increase cumulative pressure on cetacean populations if not adequately regulated. Strengthening enforcement of existing regulations, limiting the number of vessels, and promoting less intrusive technologies are therefore essential to ensure long-term ecological sustainability.

The results also highlight the potential of Marine Protected Areas as effective settings for aligning tourism and conservation objectives. When combined with high-quality interpretation and multi-stakeholder collaboration, WW activities can contribute to achieving conservation initiatives while maintaining meaningful visitor experiences. Public–private partnerships and collaboration among scientists, operators, and administrations are key to improving compliance, data exchange, and adaptive management in this field.

Finally, this study emphasises the importance of recognising the diversity of whale-watcher profiles and motivations when designing sustainable marine tourism strategies. Future research should prioritise exploring visitor segmentation based on environmental values, motivations, and perceptions of cetacean welfare. By integrating theoretical insights with practical and social considerations, this research supports WW as a viable model of sustainable ecotourism in Macaronesia, capable of balancing visitor satisfaction with the long-term conservation of marine ecosystems and the welfare of cetacean populations.

## Supporting information

S1 Appendix Expectation Questionnaire. (DOCX)

S2 Appendix Satisfaction Questionnaire. (DOCX)

S1 Fig Whale-watching participants observing cetaceans during a guided excursion in Tenerife, Canary Islands. (JPEG)
