# Supplementary material for: Do whale-watching experiences and tourist expectations align? A comparison of three Macaronesian destinations
Source: PLoS One. 2026 Mar 3;21(3):e0342997. doi: 10.1371/journal.pone.0342997 (PMC12956078; doi:10.1371/journal.pone.0342997)
Supplement: S1 Appendix — (DOCX) [file pone.0342997.s001.docx]

EXPECTATION QUESTIONNAIRE: Questionnaire on the whale watching excursion

Registration number: Data:

Locality:

Company name:

_____________________________________________________________________________________

Dear customer, we are enhancing sustainable whale watching activities through a research project in which the Cabildo de Tenerife and the University of La Laguna are participating. Your opinion and assessment are very important to us as we continue to develop it. Therefore, we ask for your collaboration in answering these questions before you leave. All data collected will be used only for our study.

Disclaimer of liability

Your participation in the questionnaire does not commit you to participating in the future. You are free to leave the questionnaire at any time. The data collected will be kept confidential, used solely for research purposes, and will not be shared with any third parties. Further information on data protection and the ULL data protection policy can be found at the following address: <https://www.ull.es/servicios/dpd/>

_____________________________________________________________________________________

1. On which island are you doing the activity?

_____________________________________________________________________________

1. On which date did you do the whale watching activity?

_____________________________________________________________________________

1. Country of residence

_____________________________________________________________________________

1. Who are you travelling with?

- Alone
- Couple
- Family
- Friends

1. What is the main reason for your trip?

_____________________________________________________________________________

1. Which species of cetaceans would you like to see on your excursion?

_____________________________________________________________________________

1. What species of cetaceans do you think you will see on your excursion?

_____________________________________________________________________________

1. What would you like to do when you encounter cetaceans?

- Quickly approach the animals
- Get as close as possible to the animals
- Stay with the animals as long as possible
- Watch them from a distance
- Feed them
- Swim with them
- Photography
- None of the above

1. On a scale of 1 (not at all important) to 5 (very important), how important is it for you to see cetaceans?


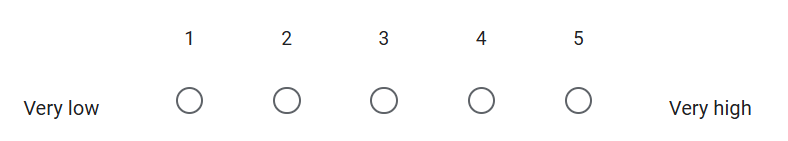


1. Is this the first time you have booked a whale watching activity?

- Si
- No

1. Did you gather enough information from our website about the species you could see on this trip?

- Yes, I think I have enough information
- Yes, but I would like to receive more information
- No, I would like more information
- No, but I don't want more information
- I don't know
