# Supplementary material for: Do whale-watching experiences and tourist expectations align? A comparison of three Macaronesian destinations
Source: PLoS One. 2026 Mar 3;21(3):e0342997. doi: 10.1371/journal.pone.0342997 (PMC12956078; doi:10.1371/journal.pone.0342997)

SATISFACTION QUESTIONNAIRE: Questionnaire on the whale watching excursion

Registration number: Data:

Locality:

Company name:

_____________________________________________________________________________________

Dear customer, we are enhancing sustainable whale watching activities through a research project in which the Cabildo de Tenerife and the University of La Laguna are participating. Your opinion and assessment are very important to us as we continue to develop it. Therefore, we ask for your collaboration in answering these questions before you leave. All data collected will be used only for our study.

Disclaimer of liability

Your participation in the questionnaire does not commit you to participating in the future. You are free to leave the questionnaire at any time. The data collected will be kept confidential, used solely for research purposes, and will not be shared with any third parties. Further information on data protection and the ULL data protection policy can be found at the following address: <https://www.ull.es/servicios/dpd/>

_____________________________________________________________________________________

1. On which island are you doing the activity?

_____________________________________________________________________________

1. On which date did you do the whale watching activity?

_____________________________________________________________________________

1. Country of residence

_____________________________________________________________________________

1. Is this the first time you have booked a whale watching activity?

- Si
- No

1. Regarding cetaceans, which animals did you expect to see?

_____________________________________________________________________________

1. Which species have you seen?

_____________________________________________________________________________

1. In general terms, what is your level of satisfaction with the excursion?


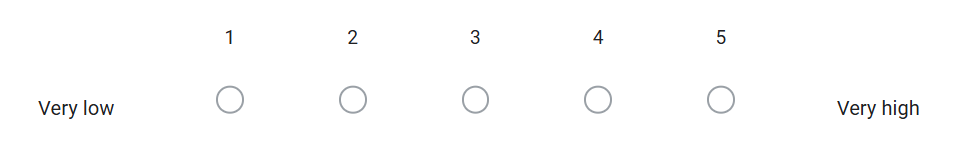

Supplement: S2 Appendix — (DOCX) [file pone.0342997.s002.docx]
