# Supplementary figures and images for: Do whale-watching experiences and tourist expectations align? A comparison of three Macaronesian destinations
Source: PLoS One. 2026 Mar 3;21(3):e0342997. doi: 10.1371/journal.pone.0342997 (PMC12956078; doi:10.1371/journal.pone.0342997)

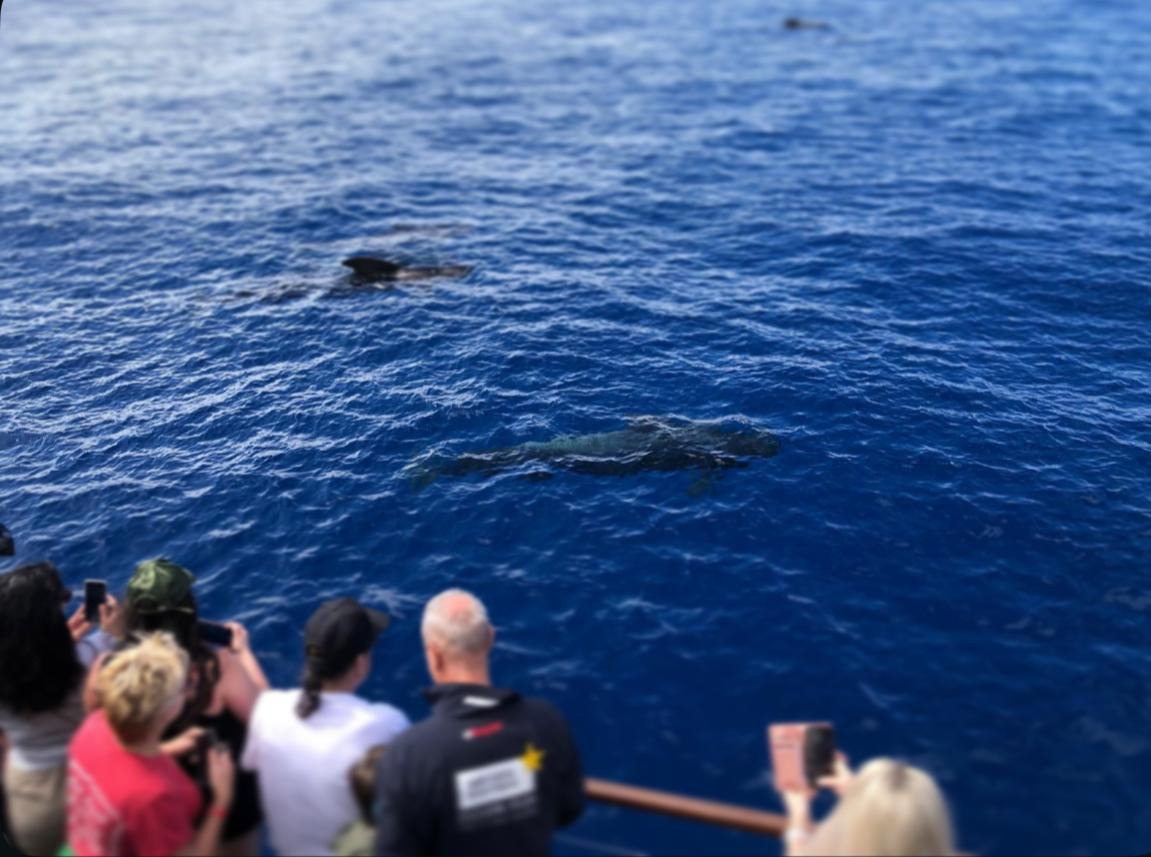

Supplement: S1 Fig — (JPEG) [file pone.0342997.s003.jpeg]
